# In Vitro Reassortment between Endemic Bluetongue Viruses Features Global Shifts in Segment Frequencies and Preferred Segment Combinations

**DOI:** 10.3390/microorganisms9020405

**Published:** 2021-02-16

**Authors:** Jennifer Kopanke, Justin Lee, Mark Stenglein, Christie Mayo

**Affiliations:** Department of Microbiology, Immunology and Pathology, Colorado State University, Fort Collins, CO 80523, USA; jennifer.kopanke@wsu.edu (J.K.); psd8@cdc.gov (J.L.); mark.stenglein@colostate.edu (M.S.)

**Keywords:** bluetongue virus, reassortment, in vitro, whole genome sequencing, amplicon-based sequencing, segmented virus

## Abstract

Bluetongue virus (BTV) is an arthropod-borne pathogen that is associated with sometimes severe disease in both domestic and wild ruminants. Predominantly transmitted by *Culicoides* spp. biting midges, BTV is composed of a segmented, double-stranded RNA genome. Vector expansion and viral genetic changes, such as reassortment between BTV strains, have been implicated as potential drivers of ongoing BTV expansion into previously BTV-free regions. We used an in vitro system to investigate the extent and flexibility of reassortment that can occur between two BTV strains that are considered enzootic to the USA, BTV-2 and BTV-10. Whole genome sequencing (WGS) was coupled with plaque isolation and a novel, amplicon-based sequencing approach to quantitate the viral genetic diversity generated across multiple generations of in vitro propagation. We found that BTV-2 and BTV-10 were able to reassort across multiple segments, but that a preferred BTV-2 viral backbone emerged in later passages and that certain segments were more likely to be found in reassortant progeny. Our findings indicate that there may be preferred segment combinations that emerge during BTV reassortment. Moreover, our work demonstrates the usefulness of WGS and amplicon-based sequencing approaches to improve understanding of the dynamics of reassortment among segmented viruses such as BTV.

## 1. Introduction

Bluetongue virus (BTV; genus *Reoviridae*, family *Orbivirus*) is associated with significant economic and animal health impacts worldwide. Composed of ten segments of double-stranded RNA (dsRNA) and transmitted by hematophagous *Culicoides* midges, BTV can cause severe disease in susceptible ruminants and has been identified as an important, re-emerging arbovirus with significant animal health implications [1,2]. BTV circulates year-round in tropical climates, and seasonally in more temperate and cooler environments [3]. Its range is defined by the presence of one or more competent vector species (*Culicoides* spp.) capable of transmitting the virus between ruminant hosts.

Recent episodes of BTV incursion into previously BTV-free regions has highlighted the role of climate change and animal and vector movements as important mediators of the spread of this virus [4,5,6]. Moreover, virus-specific factors such as mutation and reassortment may also contribute to the ability of BTV to invade new regions or cause disease outbreaks in otherwise enzootic areas. The BTV genome is replicated via an RNA-dependent RNA polymerase, which—as with other RNA viruses—lacks proof-reading ability and thus generates more replication errors during transcription [7,8,9]. For many RNA viruses, this is considered an important mechanism contributing to genetic diversification and overall fitness; the mutation rate and replication rate strike a delicate balance, allowing these viruses to rapidly adapt through the generation of large viral populations [8,10]. However, RNA arboviruses have much lower rates of mutation despite the production of large viral populations, and this is believed to be secondary to purifying selection that occur during the alternating host transmission cycle [11,12]. In vitro and in vivo studies of BTV from our lab and others indicate that this virus has a low overall rate of genetic diversification at the single-nucleotide level, and that viruses transmitted between *Culicoides* and ruminant hosts remain relatively stable at the consensus level [13,14,15].

Reassortment—the generation of progeny viruses that contain genome segments from more than one parental strain—appears to contribute significantly to the overall genetic diversification and evolution of the bluetongue virus. Extensive reassortment has been demonstrated both in vitro and in vivo with various strains of BTV, and several studies of field isolates have demonstrated that BTV reassortment is widespread in naturally transmitted viral infections [16,17,18,19,20,21,22,23]. Coinfection of the vector or ruminant host with more than one strain of BTV is a prerequisite for reassortment to occur, but thereafter, it has been suggested that reassortment has very few limitations.

Some studies—particularly those analyzing field samples—have detected potential segment-specific trends in reassortment, but most experimental approaches have generally failed to establish definitive segment combinations that are more likely to occur than others [21]. Hence, although there are certainly some trends apparent amongst field isolates, experimental validation of these findings remains to be established. The sheer number of potential segment combinations between BTV strains (2^10^ = 1024 different combinations are possible with two strains) makes the robust investigation of reassortment trends time- and labor-intensive.

Here, we apply whole genome sequencing and a novel amplicon-based sequencing approach to detect global shifts in segment frequencies and reassortment events between two enzootic North American BTV strains (BTV-2 and BTV-10) to better understand features of reassortment between these two viruses in an in vitro system. We hypothesized that BTV-2 and BTV-10 would reassort extensively, with very few limitations on the segment combinations that might arise, given a permissive in vitro environment. This coinfection system provides an interesting lens through which to assess BTV reassortment in general, as BTV-2 remains very limited in its distribution in North America, although it is considered enzootic [24,25]. Thus, the potential for this virus to reassort with other BTVs like the BTV-10 virus used here may lend important information in terms of its overall ability to spread, and could provide insight for our understanding of how incursive BTV strains may intermingle with already enzootic strains to become widespread in a new region.

## 2. Materials and Methods

### 2.1. Viruses

Two endemic bluetongue virus strains were used to established single virus infections or coinfections in vitro. BTV-2 and BTV-10 were obtained from ATCC and had been passaged three and seven times in BHK 21 cells prior to the initiation of the experiment, respectively. The BTV-2 strain was isolated from asymptomatic sentinel cattle in 1982 in Florida and subsequently submitted to ATCC (Bluetongue virus, type 2, ATCC^®^ VR-983™) [26,27]. The BTV-10 virus used in this study (Bluetongue virus, type 10, strain 8, ATCC^®^ VR-187™) was originally isolated from a sheep in California in 1952 [28].

These strains were chosen due to their enzootic nature, traceability, and distinguishability using molecular assays. Shotgun metagenomic approaches have reduced ability to distinguish highly genetically homogenous sequences, so we selected this pair of viruses due to their relatively low nucleotide identity that would allow us to clearly distinguish them using metagenomic sequencing (Table 1). The complete sequences of each virus have been deposited in GenBank (BTV-2: MW456737–MW456746; BTV-10: MW456747–MW456756).

### 2.2. Cell Culture

Low-passage BHK 21 cells were maintained in Eagle’s minimum essential medium (EMEM) with 10% heat-inactivated fetal bovine serum (FBS), 10% tryptose phosphate broth, and 1% penicillin-streptomycin (10,000 U/mL). Cells were maintained at 37 °C with 5% CO_2_ and passaged at ~90% confluency every 3–4 days.

### 2.3. Growth Curves

BTV-2 and BTV-10 were introduced in duplicate to confluent cultures of BHK 21 cells at a multiplicity of infection (MOI) of 0.2 TCID50/mL. A negative control flask inoculated with an equal volume (1 mL) of EMEM without virus was also included. Viral inoculum was allowed to incubate with cells for 1 h at 37 °C, and then an additional 4 mL of maintenance media was added to each flask. Five hundred µL of cell culture supernatant was collected at 1 h, 6 h, 12 h, 24 h, 48 h, and 72 h post-inoculation and stored at −80 °C until further analysis. Tissue culture infectious dose (TCID50) was calculated from the supernatant collected at each time point to characterize the viral growth kinetics of each virus.

### 2.4. Viral Passages

Following initial virus characterization, BTV-2 and BTV-10 were used to inoculate flasks of confluent BHK 21 cells in triplicate. To establish single-virus infections, the virus was diluted in EMEM to reach a concentration of 0.2 TCID50/mL. One ml of diluted virus was then added to a confluent monolayer of BHK 21 cells in triplicate. To establish coinfections, each virus was diluted to a final concentration of 0.2 TCID50/mL in EMEM, and 1 mL of inoculum containing 0.1 TCID50/mL of each virus was added to confluent flasks of BHK-21 cells in triplicate. Another flask of cells was inoculated with EMEM as a negative control. After 1 h of incubation at 37 °C, an additional 4 mL of maintenance media was added to each flask.

The virus was harvested from cell cultures at 72–96 h post-inoculation, when the cytopathic effect (CPE) was approximately 80–90%. Freshly harvested virus was used to initiate the next round of infections on BHK 21 cells immediately following collection. Viruses were passaged blindly so as to avoid freeze-thaw cycles. Three hundred µL of virus was also reserved to perform TCID50s at each passage. Remaining harvested virus was aliquoted into 1 mL vials and stored at −80 °C for downstream analysis.

### 2.5. TCID50

TCID50s were performed for viral quantification to determine initial MOI, viral titers during growth curves, and levels of infectious virus at each passage throughout the course of the study. Briefly, ten-fold dilutions of virus stocks were prepared in triplicate from 10^−1^ to 10^−8^. Fifty µL of each dilution of virus (or EMEM for negative controls) was added to a well in a 96-well microtitration plate. BHK 21 cells were seeded at a density of 1.55 × 10^4^ cells per well, along with 50 µL of EMEM. Fifty µL of maintenance medium was added to each well at 24 and 72 h post-inoculation, and plates were stained with crystal violet solution and read at 96 h. The Reed–Muench equation was used to determine the TCID50/mL for each virus [29].

### 2.6. RT-PCR

Nucleic acid was extracted either manually or on the KingFisher Flex robot (Thermo Fisher, Waltham, MA, USA) using Applied Biosystem’s MagMAX Pathogen RNA/DNA kit (Foster City, CA, USA) according to manufacturer’s instructions.

A pan-BTV RT-PCR assay targeting segment 10 was performed as described by Hofmann et al., with modifications as outlined by Ortega et al. [30,31] RT-PCR was performed using SuperScript III Platinum One-Step qRT-PCR reagents (Invitrogen, Carlsbad, CA, USA) at half-reaction volumes, and thermocycling conditions were carried out as previously described [30,31]. Samples from each passage were screened throughout the course of the study.

### 2.7. Whole Genome Sequencing

Samples from passages 1, 3, and 7 were prepared for shotgun metagenomic sequencing. Samples were DNased according to manufacturer’s instructions using TURBO DNA-free kit (Invitrogen), except the amount of DNase was increased to 4U per sample to maximize DNase activity. Following DNase inactivation, 7.5 M LiCl solution was added to each sample to reach a final concentration of 2.0 M. Samples were incubated for 16–18 h at 4 °C, then centrifuged at 18,000× *g* at 4 °C for 20 min to selectively precipitate single-stranded RNA. Supernatant was subsequently used for downstream whole genome sequencing (WGS). Sample quality was determined with RNA High Sensitivity screentape on a TapeStation 2200 instrument (Agilent, Santa Clara, CA, USA) to estimate RNA integrity and concentration prior to library preparation.

Sample libraries were prepared using KAPA RNA Hyper Prep kit (KAPA Biosystems, Basel, Switzerland) according to instructions, except reagents were used at half-reaction volumes with 5 µL of sample input. Libraries were assessed for DNA quality and concentration using Qubit broad-range or high sensitivity DNA reagents and the Qubit 2.0 fluorometer (Thermo Fisher, Waltham, MA, USA), followed by High Sensitivity D1000 DNA screentape on a TapeStation 2200 machine. Samples were then pooled and size-selected for inserts from 300–800 basepairs (bp) in length using size fractionation on a 1% agarose gel. The desired region was excised from the gel and a QIAquick Gel Extraction kit (Qiagen, Hilden, Germany) was used to purify the pooled DNA according to manufacturer’s instructions.

Pooled, size-selected libraries were then re-analyzed using Qubit fluorometer and the TapeStation 2200 system as before. The pooled library was quantified further using KAPA Library Quantification kit (KAPA Biosystems) according to kit instructions, prior to loading on the flowcell. Libraries of approximately ~20 samples were sequenced using NextSeq 2 × 150 mid-output reagents (Illumina Inc., San Diego, CA, USA) on an Illumina NextSeq machine.

### 2.8. BTV Analysis Pipeline and Bioinformatics

Consensus sequences of BTV-2 and BTV-10 input strains were determined via our lab’s novel BTV bioinformatics pipeline, as previously described [15]. Viral supernatant harvested from passages 1, 3, and 7 was similarly processed, with some minor changes. Briefly, libraries were demultiplexed and raw reads were trimmed to remove low quality bases and adapter sequences [32]. Cd-hit was then used to eliminate duplicate reads (two or more reads with ≥96% pairwise identity in the first and last 30 base pairs) [33]. Processed reads were aligned to consensus sequences of the input viruses, BTV-2 and BTV-10, using Bowtie2 [34]. Bowtie2 default parameters were used, except end-to-end and very sensitive parameters, which were specified to reduce off-target mapping [34]. Sequences were visually inspected in Geneious v. 10.2.2 to ensure alignment and mapping accuracy.

### 2.9. Plaque Isolation

To identify the genotype of individual plaque forming units and to detect potential occurrences of reassortment, viral stocks harvested from passages 1, 3, and 7 co-infected cultures were serially diluted and used to establish infections on 6-well plates of BHK 21 cells. Six-well plates were prepared 48 h prior to virus inoculation, with each well seeded with 1.0 × 10^5^ cells in maintenance media.

Serial ten-fold dilutions of viral stocks from 10^−1^ to 10^−8^ were prepared in EMEM. Five hundred µL per well of 10^−2^ to 10^−7^ viral dilutions were used to inoculate confluent BHK 21 monolayers in duplicate. After 1 h of incubation at 37 °C, cells were washed once with PBS to remove unbound virus and then were overlaid with 2 mL of a 1:3 solution of 2% agarose in Earle’s balanced salt solution (EBSS): maintenance media. Plates were incubated at 37 °C, with 5% CO_2_ for 48–72 h, when cytopathic effect (CPE) was evident. Once CPE was detected, a second overlay containing 0.1% neutral red in media with agarose was added, after which cells were incubated until discrete plaques were visible.

Plaques were visualized using a transilluminator or by holding the plates up to a light. Individual plaques were picked and propagated once in BHK 21 cells in a 24- or 48-well plate format. Briefly, each agarose plug was added to 100 µL EMEM and 4.65 × 10^4^ BHK 21 cells in 300 µL maintenance media in a well, and propagated plaques were maintained at 37 °C until CPE was complete. Viral supernatant was harvested, aliquoted, and then frozen at −80 °C until downstream analyses were carried out.

### 2.10. Amplicon Assay

Total nucleic acid from propagated plaques was extracted either manually or on the KingFisher Flex robot using Applied Biosystem’s MagMAX Pathogen RNA/DNA kit as described above. Extracted RNA was subsequently used for amplicon-based sequencing to rapidly differentiate which parental strain contributed each of the ten segments comprising the progeny virus. Standards and negative controls were run in duplicate with each plate of samples. Standards were prepared from BTV-2 and BTV-10 stocks with equal Ct values (based on segment 10 RT-PCR; see above) as follows: standard 1, 100% BTV-2; standard 2, 90% BTV-2/10% BTV-10; standard 3, 50% BTV-2/50% BTV-10; standard 4, 10% BTV-2/90% BTV-10; standard 5, 100% BTV-10. These standards were used to ensure we could reliably and specifically detect all ten segments of each virus, as well as establish baseline levels of any off-target mapping that might occur for each virus and segment.

Custom primers were used to generate barcoded amplicons containing Illumina’s TruSeq adapter sequences from each BTV segment following the two-step PCR assay design described by Galan et al [35]. Full-length viral genome sequences of each parental strain were aligned and used to design consensus primers for each segment using the Primer3 plugin in Geneious v.10.2 [36]. First round primers were designed to generate amplicons approximately 400–600 bp in length that were genetically distinct between BTV-2 and BTV-10 at multiple sites within the amplified region. These first round primers included two main features: a BTV segment-specific primer region and a 5′ adapter sequence complementary to the second round primer set (Supplemental Materials Table S1). Eight base pair dual-indexes were generated for forward and reverse round-two primers (96 unique indexes each) using BARCRAWL (Supplemental Materials Table S1) [37]. Individual round-one primer pairs were confirmed to amplify their specific target in each parental genome and pooled equimolar into a 10-plex reaction. An initial test-run of the multiplex PCR with the standard samples (above) was sequenced in the Illumina MiSeq (Illumina, Inc.) and the concentration of each primer pair was adjusted empirically based on the relative efficiency of each primer pair (proportion of reads for each product).

The first round PCR included an initial template denaturation and primer annealing step with 2 µL of sample and 2 µL of 2 µM first-round primer pool heated at 95 °C for 5 min and then immediately quenched on ice. The following master mix using components from SuperScript III One-step RT-PCR kit with Platinum Taq (Invitrogen) was then prepared and added to each reaction on ice for reverse transcription and round-one amplification: 5 µL of 2X Reaction Mix, 0.5 µL SuperScript III RT/Platinum Taq mix, 0.5 µL H2O. Thermocycling conditions were as follows: 56 °C × 30 min → 94 °C × 2 min → 14–16 cycles of [94 °C × 15 s + 54 °C × 45 s + 68 °C × 30 s] → 68 °C × 30 s. Round-one product was treated with Exonuclease 1 from New England Biosciences (Ipswish, MA, USA) according to kit instructions to remove excess primers.

The second round PCR included 1 µL each of forward and reverse barcoded primers (2 µM), in addition to a master mix containing the following components: 1 µL each of i5 and i7 primers (Supplemental Materials Table S1), 0.1 µL Platinum Taq High Fidelity DNA Polymerase (Invitrogen), 3.7 µL H_2_O, 1 µL 10× Reaction Buffer, 0.7 µL MgCl2, and 0.5 µL 10 mM dNTP mix. PCR reaction conditions were as follows: 94 °C × 2 min → 18–20 cycles of [94 °C × 30 s + 52–54 °C × 45 s + 72 °C × 30 s] → 72 °C × 30 s.

Second-round product was quantified using fluorometric quantification with Sybr Green (Thermo Fisher) on a plate reader (EnSpire Multimode Plate Reader, PerkinElmer, Waltham, MA, USA). Up to 96 products were then pooled to approximately equimolar concentrations and purified with Agencourt AMPure XP beads (Brea, CA, USA) (0.6× ratio). The pooled amplicon library was quantified on the Qubit 2.0 fluorometer and visualized using the Agilent TapeStation 2200 prior to KAPA qRT-PCR quantification. Library pools were sequenced on an Illumina MiSeq instrument using either 500 cycle Nano or 300 cycle Micro kits.

### 2.11. Amplicon Assay Bioinformatics

Illumina reads were demultiplexed with bcl2fastq v2.20.0.422 using default settings [38]. Primer and adapter sequences were trimmed using Cutadapt v1.13, and 3′ bases were removed if they were below a minimum quality of Q30 [39]. Reads less than 80 bp in length after trimming were removed from further analyses. Trimmed reads were mapped to the parental reference sequences in Bowtie2 v2.3.2 (Supplemental Materials Table S2) [34]. Sorted bam files were made in samtools v1.5 and viewed in Geneious v.10.2.2 to confirm accurate mapping of reads to the correct parental strain(s) [40]. The reads mapping to each parental strain were quantified and used to determine the presence of reassortment in progeny viruses (presence of reads mapping to one or more segments from both parental strains). Only plaques with >90% of all reads mapping to one parent segment or the other were included in our final analyses. Viruses with missing segments (i.e., those that did not get reads across all ten segments) or those that had reads for one or more segments that mapped to both BTV-2 and BTV-10 were also excluded from downstream analyses.

### 2.12. Relative Diversity

Simpson’s diversity index was used to estimate the population complexity of viruses isolated from co-infected conditions. This measure is based upon the number of unique genotypes (richness) present in a population, as well as the relative abundance of each (evenness) [41]. Simpson’s diversity was calculated for each BTV-2+10 replicate from passages 1, 3, and 7 using the following equation: $D\;=\sum^{\;}\left(p_{i}{}^{2}\right)$ where *p_i_* is the relative abundance of each genotype. To normalize these data to a linear format and to allow for more intuitive comparisons, *D* for each sample was converted to Hill’s N_2_, which can be calculated by $N_{2}\;=\;1/D$ [42]. Hill’s N_2_, or the effective diversity of the population, represents the number of equally abundant genotypes required to be present to generate the level of diversity detected by Simpson’s index [42,43].

### 2.13. Statistics

Chi-square analysis and Fisher’s exact test were performed to determine whether significant trends existed in terms of segment-linkages and reassortment for amplicon-based genotypes, with *p* < 0.05 considered significant. Two-way repeated measures ANOVA with Tukey’s post hoc was used to determine whether significant trends in segment frequencies from metagenomic data occurred across passages, with *p* < 0.05 considered significant. Statistical analyses were carried out with GraphPad Prism v. 8.0.

## 3. Results

### 3.1. Viral Growth Curves

**Figure 1:**
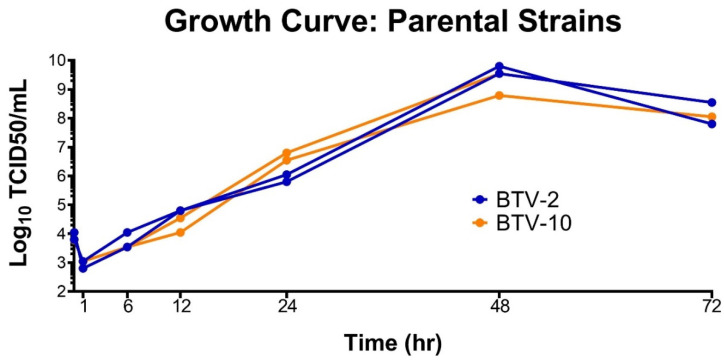
Growth Curves of Parental Strains. BTV-2 and BTV-10 demonstrate similar growth kinetics in BHK 21 cells. BTV-2 or BTV-10 was used to initiate single-virus infections in duplicate on BHK 21 cells with a starting MOI of 0.2 TCID50/mL. Supernatant was collected at 1, 6, 12, 24, 48, and 72 h post infection and TCID50s were performed at each time point to determine infectious titer.

To ensure we did not unfairly bias our coinfection experiment with one virus growing more rapidly than the other, we performed growth curves for both BTV-2 and BTV-10 in BHK 21 cells prior to initiating coinfections. Growth curves demonstrated that BTV-2 and BTV-10 had similar growth kinetics in the target cell type (BHK 21) (Figure 1).

Infectious titer and viral copy number of each replicate were tracked across each passage by TCID50 and RT-PCR, respectively, and remained relatively constant between replicates and infection conditions (single vs. coinfection) (Supplemental Materials Figure S1).

### 3.2. Whole Genome Sequencing

Previous studies have reported that BTV remains largely genetically stable across passages in vitro and during experimental transmission in vivo [13,15]. To understand whether this phenomenon also occurs in BHK 21 cells, which are interferon-deficient and hence may provide a permissive environment for genetic diversification, or whether coinfection might drive the occurrence of mutations, we assessed the consensus sequences of BTV-2, BTV-10, and BTV-2+10 at the beginning (passage 0, input viruses) and the end of our passage series (passage 7, output viruses), using shotgun whole genome sequencing (WGS) [44]. Consensus sequences were constructed for each segment and coding sequences of passage 0 viruses were aligned to those of passage 7 viruses. For certain segments from one parental virus or the other (Table 2), full coding sequences could not be determined due to the very low frequency of these segments in co-infected cultures by passages 3 and 7. There were no consensus changes detected between passages 0 and 7, regardless of infection condition (single vs. coinfection).

As has been performed for influenza virus, WGS was used to detect global shifts in population segment frequencies during BTV coinfection as an estimator of reassortment trends [45]. Viral supernatant collected from each BTV-2+10 replicate after passages 1, 3, and 7 was assessed via WGS to determine the segment composition of the viral population present at each time point.

Reads from each sample were aligned to both BTV-2 and BTV-10 via our sequencing pipeline [15]. The number of reads per segment aligning to either parent strain was normalized as a percent by the total number of reads per segment.

**Figure 2:**
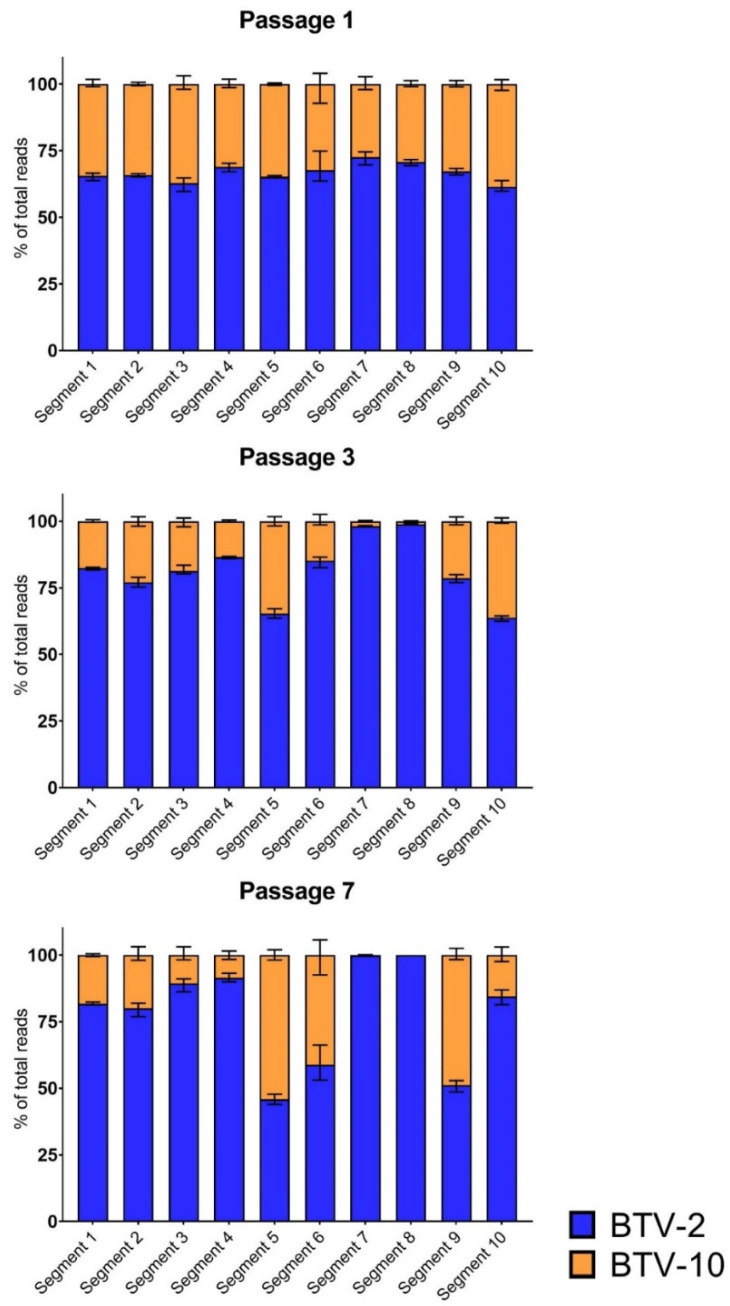
Whole Genome Sequencing Trends Across Passages. Metagenomic sequencing shows distinct trends across segments 1–10 during BTV-2+10 coinfection over seven passages. The percentage of total viral reads aligning to either input strain (BTV-2 in blue; BTV-10 in orange) are shown for each segment for passages 1, 3, and 7. Error bars depict mean and range.

Following a single passage in BHK 21 cells, viral populations from BTV-2+10 coinfections demonstrated early, consistent changes in overall segment composition. While all ten segments from both BTV-2 and BTV-10 were present and well-represented after one passage, all replicates demonstrated a consistent shift towards BTV-2, with approximately ⅔ of reads for each segment aligning to BTV-2, and only ⅓ of reads aligning to BTV-10. This trend occurred across all ten segments in each replicate (mean: 67%, range 60–75% BTV-2) (Figure 2).

By the third passage, marked trends in segment frequencies were more evident, with prominent shifts towards BTV-2 across most segments (Figure 2). This was most remarkable for segments 7 and 8, where >98% of reads aligned to BTV-2. Segments 5 and 10 were notable exceptions to this trend, however, maintaining relatively substantial contributions from both BTV-2 (~65% of viral reads) and BTV-10 (~35% of viral reads) within the viral population present at passage 3.

In general, the trends in segment contributions from each BTV parent strain noted at passage 3 persisted after seven passages, with shifts towards BTV-2 becoming more pronounced across most segments. No BTV-10 reads were detected for either segment 7 or 8 in two of three replicates. In one replicate, 0.2% of segment 7 reads aligned to BTV-10. BTV-2 and BTV-10 contributed roughly equivalent proportions of segment 5 reads, while segment 10 shifted heavily towards BTV-2 (84% of viral reads). In contrast to passage 3, segment 9 demonstrated substantial representation of both parent strains in passage 7.

Two-way repeated measures ANOVA with Tukey’s post-hoc to correct for multiple comparisons was used to determine whether shifts in segment frequencies along the course of passages were statistically significant. The overall contribution of BTV-10 segments 1, 2, 3, 4, 7, 8, and 10 significantly decreased from passage 1 to passages 3 and 7 (all *p* < 0.05). The percentage of reads aligning to BTV-10 segment 9 was significantly increased from passages 1 and 3 to passage 7 (*p* < 0.05). No significant differences were detected in the fraction of reads aligning to BTV-10 segment 6 across passages.

### 3.3. Amplicon-Based Sequencing

We used a novel, amplicon-based sequencing assay to rapidly differentiate the genotypes of individual viral plaques isolated from passages 1, 3, and 7. Standards composed of known quantities of BTV-2 and BTV-10 were used to ensure that all 10 segments from each virus were successfully detectable and distinguishable using this approach (Supplemental Materials Figure S2). There was no cross-mapping to the other genotype when pure populations of BTV-2 or BTV-10 were sequenced. In the mixed standards, the fraction of reads mapping to each genotype varied in the different segments. Some segments were over- or under-represented relative to expected fractions, consistent with differential PCR efficiencies for the different segments. This indicated that this amplicon assay would be best suited for assessing the genotypes of individual plaques, and less well suited for accurately quantifying the abundance of different segments in a population.

**Figure 3:**
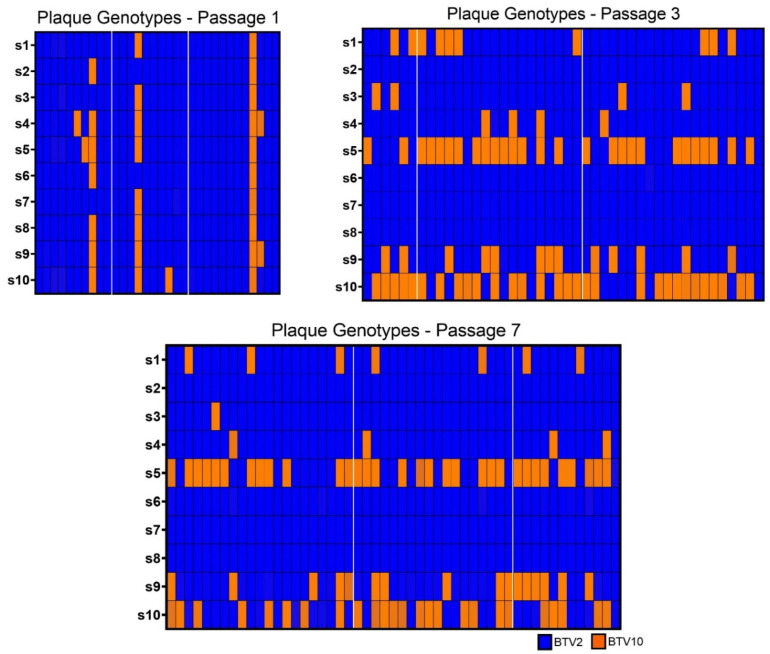
Plaque Genotypes. Plaque genotypes from isolates collected at passages 1, 3, and 7 were determined using amplicon-based sequencing to detect the identify of each segment. Segments derived from BTV-2 are shown in blue, while those derived from BTV-10 are shown in orange. The segment identities of each isolate for segments 1 through 10 (s1, s2, s3, s4, s5, s6, s7, s8, s9, s10) are depicted in descending order in each column so that each column represents the full ten segments of an individual plaque (i.e., its complete genotype). Plaques isolated from each replicate at passages 1, 3, and 7 are demarcated by white margins.

We successfully sequenced 32 plaques from passage 1, 44 plaques from passage 3, and 51 plaques from passage 7 that had a clear genotype across all ten segments (>90% of reads aligning to one parent strain or the other) without evidence of plaque bleed-over or plaques that were seeded by >1 particle (Figure 3). Similar to our population level WGS results, we found that no plaques had either segment 7 or 8 contributed by BTV-10 in passages 3 and 7, and that both parental strains of BTV were well-represented across plaques in segments 5 and 9.

**Figure 4:**
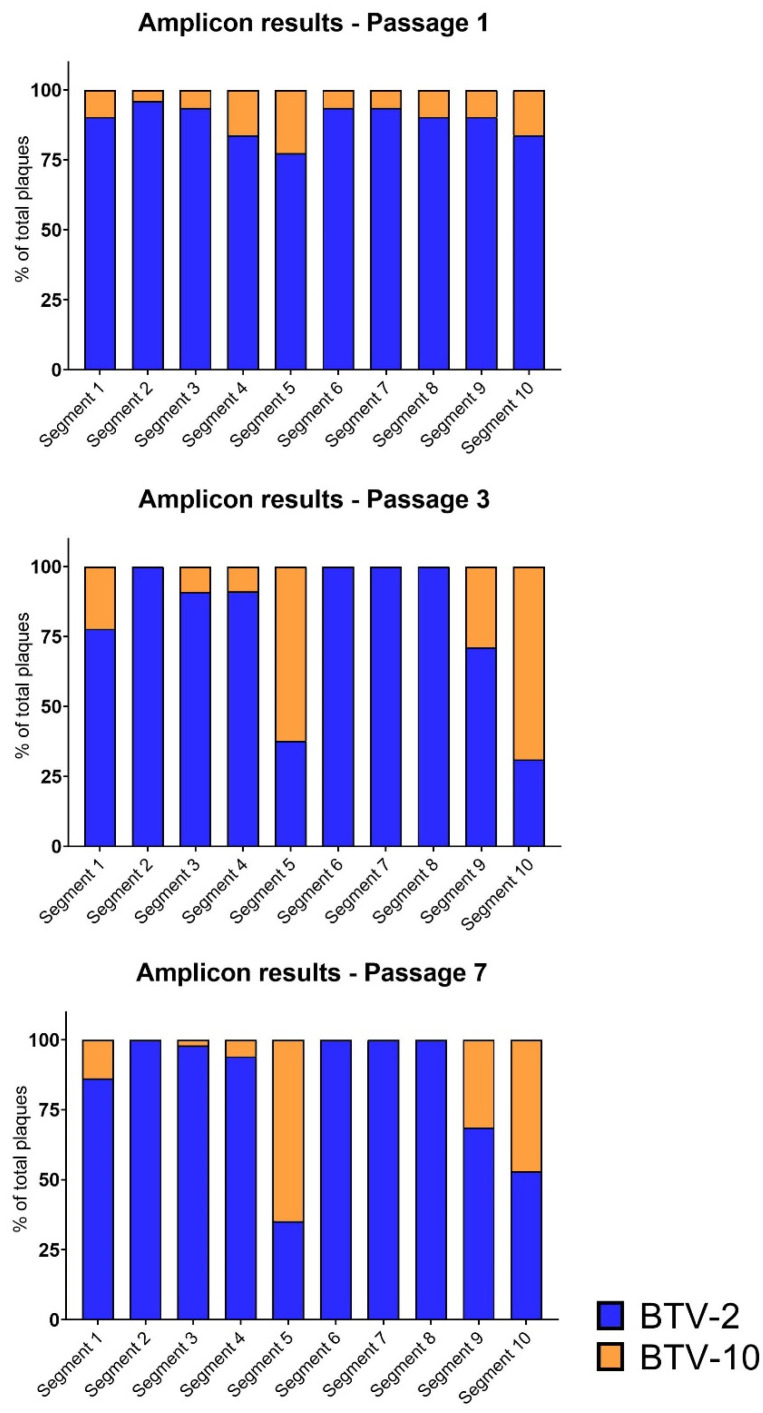
Segment Frequencies Based on Amplicon-based Genotyping. Data as in Figure 3 but aggregated for all plaques. Amplicon-based genotyping demonstrates similar trends to metagenomic sequencing, but shows unique trends for certain segments as detected via plaque assays. Cumulative data across all coinfection replicates were used to create relative percentage of plaques with each segment represented by either input virus (BTV-2 vs. BTV-10) at passage 1, 3, and 7. The percentage of plaque isolates with a segment contributed by BTV-2 is shown in blue, while the percentage of plaque isolates with a segment contributed by BTV-10 is shown in orange.

Interestingly, although BTV-10 contributed only ~⅓ of segment 10 viral reads in passage 3 and ~⅕ of segment 10 viral reads in passage 7 via metagenomic sequencing, we detected a high frequency of plaques with BTV-10 segment 10 by our plaque assay method (Figure 4). We also found that certain segments that were well-represented in our metagenomic sequencing data were not detected by our plaque assay approach; in particular, BTV-10 contributed approximately 40% of segment 6 viral reads in passage 7 according to WGS, but not a single plaque with BTV-10 segment 6 was identified. A similar, although less striking, trend was noted for BTV-10′s segment 2. It is important to note that these two “missing” BTV-10 segments were detectable in our amplicon assay positive control standards, as well as in propagated plaques that did not have clear genotypes or appeared to have plaque bleed-over, indicating that viruses with these BTV-10 segments may have had slower growth kinetics or diminished cytopathic effect on BHK 21 cells.

### 3.4. Simpson’s Diversity Index

**Figure 5:**
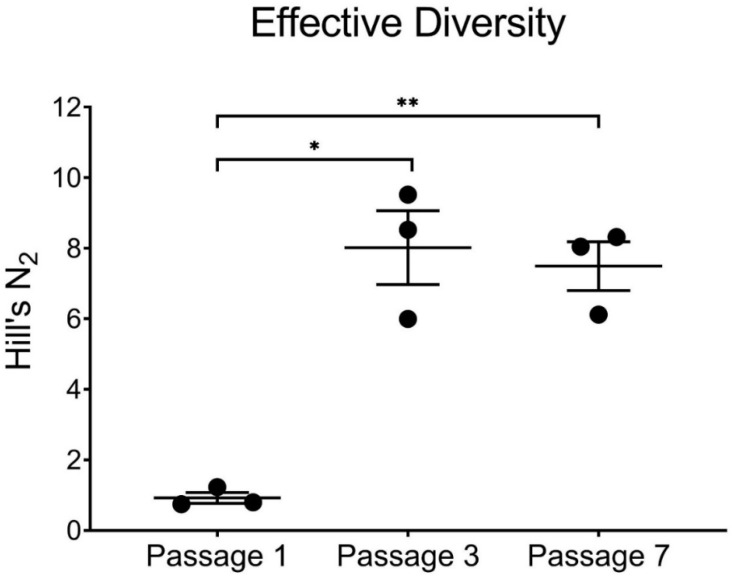
Effective Diversity across Passages. Hills N_2_ and effective diversity are increased by passages 3 and 7 in co-infected cultures (* *p* < 0.05, ** *p* < 0.01, paired t-test). Each replicate is depicted by a black circle, with mean and standard deviation error bars shown for each passage.

To better understand the genetic diversity generated by BTV-2 and BTV-10 coinfection, we calculated Simpson’s diversity index and Hill’s N_2_ for each replicate from passages 1, 3, and 7 [41,42,43]. While all 20 potential segments from BTV-2 and BTV-10 were represented after the first passage, effective diversity was actually quite low, due to the large number of plaques that had all ten segments contributed by BTV-2. Although certain BTV-10 segments all but disappeared from co-infected viral populations by passages 3 and 7, effective diversity nevertheless was increased from passage 1 as a result of increased numbers of reassortant genotypes (Figure 5).

There were 8 unique genotypes identified in passage 1, 17 in passage 3, and 17 in passage 7 (across all replicates). While the number of unique reassortant viruses was relatively high in passages 3 and 7, there were consistent trends in terms of which segments were involved in reassortment events (segments 5, 9, 10 frequently; segments 1, 3, and 4 infrequently), and these were noted in each replicate. In general, most isolated viruses appeared to have a BTV-2 backbone consisting of BTV-2 segments 2, 3, 4, 6, 7, and 8, that occasionally accepted certain BTV-10 segments, especially BTV-10 segments 5, 9, and 10 (Figure 3).

## 4. Discussion

Reassortment amongst bluetongue viruses is a complex phenomenon, and our understanding of the features that drive or restrict the occurrence of reassortment remains limited. Here, we applied whole genome sequencing and a novel, amplicon-based sequencing approach to characterize the occurrence of reassortment between two endemic bluetongue viruses in a relaxed, in vitro system.

We selected BHK 21 cells as our model system for this particular study for two reasons. First, both BTV-2 and BTV-10 grow similarly in BHK 21 cells, allowing us to avoid unfairly biasing our experiment with one virus simply outcompeting the other. It is important to note, however, that initial BTV-2 and BTV-10 growth curves were performed using single-virus infections, so it remains possible that these viruses interfere with each other’s replication in a coinfection scenario. Second, BHK 21 cells are interferon-deficient, and therefore provide a relaxed environment for BTV replication [44]. We sought to understand—at its most basic level—how readily reassortment occurs between these two viruses, so we used a permissive in vitro system to simply query the extent to which these viruses can exchange compatible segments.

Our findings highlight the plasticity with which BTV may reassort, given the appropriate conditions. This corroborates the findings of Shaw et al., who investigated reassortment between BTV-1 and BTV-8 in an in vitro system and found that the viruses reassorted extensively [46]. Our work also correlates to the findings of a much earlier series of reassortment studies where BTV-10 (BT-8) and BTV-17 (strain 262) coinfections were investigated in Vero cells and in vivo using shifts in electropherotype as a hallmark of reassortment [16,17,18,20]. These studies found that reassortment occurred readily between BTV-10 and BTV-17 viruses, both in vitro and in vivo.

In their 2013 study, Shaw et al. noted potential trends in terms of which segments were most likely to be detected in reassortant viruses (segments 1, 2, and 7 donated by BTV-8, for instance) after four passages [46]. However, this group concluded overall that reassortment was highly flexible and no specific trends were apparent during BTV-1 and BTV-8 coinfection. In contrast, we found consistent, repeatable trends in segment frequencies, both in our metagenomic sequencing approach and when we assessed the specific composition of individual viruses. These trends were noted after only three passages in BHK 21 cells, and became even more pronounced—although with slight shifts—by passage 7. We even found that some segments went “extinct” in our reassortment experiment, and compellingly, all replicates demonstrated highly similar trends at each time point. This suggests that there may be specific, preferred segment combinations that arise during BTV-2 and BTV-10 reassortment, favoring a BTV-2 backbone (segments 2, 3, 4, 6, 7, and 8) that more readily accepts certain segments from BTV-10. These preferred segment combinations may be a function of the particular pair of coinfecting parental BTVs.

In assessing the specific trends that we detected in both our metagenomic sequencing approach and our amplicon-based genotyping assay, one of the most striking findings was that BTV-10 segments 7 and 8 essentially disappeared from the viral population over the course of our BTV-2+10 passage series. Remarkably, this occurred despite 100% shared identity at the amino acid level of the protein coding region between BTV-2 and BTV-10′s segment 7 (VP7 protein). The concurrent disappearance of both of these segments, despite identical BTV-2 and BTV-10 VP7 proteins, suggests potential interactions between segments 7 and 8 during viral replication, either at the RNA-RNA level or at the protein-RNA level. Alternatively, the disappearance of BTV-10 segments 7 and 8 may reflect a mismatch between these segments and an increasingly frequent BTV-2 backbone. It is also possible that BTV-2′s segments 7 and 8 are selectively incorporated during viral replication and packaging. While segment 8′s NS2 protein is known to be an essential component of viral inclusion bodies (VIBs) and plays a key role in recruiting and binding viral ssRNAs for assembly, direct NS2-segment 7 interactions have not been described to date [47,48].

These findings are also important because they allude to specific segment linkages that might limit the spectrum of fit reassortant viruses that can be generated during BTV coinfection. When Shaw et al. generated mono-reassortants with segments from BTV-1 or BTV-8 onto the reciprocal virus’s backbone, they found deleterious effects when BTV-8 segment 7 was incorporated into the BTV-1 backbone [46]. They also found that segment 8 mono-reassortants demonstrated consistently decreased titers 8 h post infection compared to wild-type viruses. Collectively, these findings suggest that segment 7 and 8 mismatches may negatively affect viral fitness.

We also found that there was a progressive disappearance of BTV-10 segment 3 across passages. It is possible that this trend is linked to the disappearance of BTV-10 segment 7, as the VP3 and VP7 proteins—encoded by segments 3 and 7, respectively—interact structurally to form the inner core of the virion. As a result, it is possible that structural incompatibilities between BTV-2 and BTV-10′s VP3 and VP7 proteins are responsible for the near-disappearance of BTV-10 segment 3 from the viral population.

Interestingly, BTV-2 and BTV-10 demonstrated distinct cytopathic effect (CPE) phenotypes during infection, with BTV-10 causing profound, rapid cell lysis, and BTV-2 resulting in less profound lysis and comparatively prominent cell rounding and cytomegaly. BTV-2+10 replicates increasingly adopted this BTV-2-like CPE phenotype during the passage series. The BTV-2-like CPE phenotype observed during coinfection may point to the complete incorporation of BTV-2 segment 8 and disappearance of BTV-10 segment 8 by passage 7, as NS2 (encoded by segment 8) has been found to play a key role in disrupting mitosis in BTV-infected cells [19]. Certain strains of BTV have been linked to aberrant mitosis in BHK 21 cells and other cell types (Vero, BPAEC), which was associated with NS2 accumulation near kinetochores [19]. Thus, the shift in phenotype observed during BTV-2+10 coinfection may reflect specific properties of the BTV-2 segment 8 protein, as it interacts with cell machinery during mitosis, which may represent an adaptive advantage of BTV-2′s segment 8 (at least for in vitro propagation).

Metagenomic sequencing also revealed that certain segments from BTV-10 were more likely to reassort onto a BTV-2 backbone than others. Specifically, we saw high frequencies of both BTV-2 and BTV-10 contributing segments 5, 6, and 9 in passage 7 co-infected conditions. Segment 5 encodes the NS1 protein, which is associated with microtubules and has been shown to enhance BTV translation; segment 6 encodes VP5, one of the two major outer capsid proteins; and segment 9 encodes VP6 and NS4 [49,50]. VP6 is the RNA helicase, while NS4 is translated from another open reading frame and acts as an interferon antagonist [51,52]. Specific interactions between these three segments have not been described, nor do these segments share the highest nucleotide or amino acid identity of the ten segments between the BTV-2 and BTV-10 used in our study. At least for segments 5 and 9, it appears that a BTV-2 backbone virus can accept these segments from either parental strain successfully.

As BTV-10 segment 6 was not identified in any of our plaques, it is difficult to draw conclusions about possible linkages between this segment and others. This highlights another key finding from our study—namely, that plaque assays failed to fully capture the genetic diversity present in our viral populations as detected by WGS. Conversely, the composition of the BTV population was not an accurate predictor of the subset of segment combinations that could successfully form an infectious particle and seed a plaque. While individual plaque genotypes largely corresponded with metagenomic sequencing findings, we found that certain segments that were present at high levels by whole genome sequencing (e.g., BTV-10 segment 6) were not detected among our plaque-isolated viruses. Not only does this emphasize the importance of whole genome sequencing in capturing viral population composition, but it also demonstrates that complete reliance upon plaque assays to understand reassortment may fail to detect important trends in segment frequencies.

It is possible that our WGS results reflect RNA from non-infectious BTV particles that are carried along from passage to passage and possibly replicated via complementation. Only plaques that had >90% of reads aligning to one parental segment or the other were included in our amplicon assay analysis, so plaques that did not meet this criterium were excluded. However, a recent study on the Zika virus found that a large proportion of plaque-forming units were composed of multiple viral particles, and collective transmission of viral genomes has been reported for several viruses, including rotavirus [53,54]. Therefore, it is possible that the discrepancies noted between WGS and our amplicon results are due to the exclusion of amplicon data from plaques with reads for one or more segments aligning robustly to both parental strains.

We also found an overrepresentation of reassortant isolates with BTV-10 segment 10. While our WGS data indicated that BTV-10 segment 10 was actually the minor allele present in the population, selection of individual plaques seemed to disproportionately favor plaques with BTV-10 segment 10. In early experiments based on electropherotype using BTV-10 (BT-8) and BTV-17 (strain 262) in Vero cells, Ramig et al. found a higher than expected number of reassortant plaques with BTV-10 segment 10, even when BTV-10 was added at a lower MOI [18]. They also found that segment 8 was significantly more likely to be contributed by the opposite virus (BTV-17). Similar findings were noted in *Culicoides variipennis* (now *sonorensis*) coinfections [17,18].

The NS3/3a protein, encoded by segment 10, is associated with the development of CPE in mammalian cells, due to—among other factors—its viroporin-like activity [55]. Some groups have shown that single-amino acid residue changes in NS3 affect the development of CPE in vitro, as well as the virulence of BTV in IFNAR^−/−^ mice [56]. The frequent detection of plaques with BTV-10 segment 10 in our study and others, therefore, may be related to a more dramatic viroporin activity and CPE phenotype generated by viruses incorporating BTV-10 segment 10 compared to BTV-2′s segment 10.

Importantly, the MOI that we used for coinfection (0.1 TCID50 of each virus) may have impacted the occurrence of reassortment and the segment-specific trends that we detected. For other segmented viruses such as influenza, higher MOIs are linked with increased frequency of reassortment, so we may have detected different trends had we used a different MOI [57]. Furthermore, different ratios of each parental strain of virus—as would likely be seen in naturally occurring cases of coinfection—could also affect the generation, fitness, and selection of reassortant progeny viruses. The biological relevance of very high MOIs for either mammalian or insect BTV infection is questionable, given the small volumes of blood ingested and expectorate released during *Culicoides* feeding. However, repeated experiments with higher MOIs or different ratios of virus are warranted to investigate this phenomenon further.

In terms of the broader ecological relevance of this work, certain details are of note, particularly in reference to our selection of BTV-2 as one of the two strains used here. BTV-2 was first detected in the U.S. in 1982 in Florida [26]. It has been infrequently identified on subsequent occasions, most commonly in Florida and the southeast [24,58,59]. In 2010, BTV-2 was isolated from a dairy heifer in California, leading to concerns about the expansion of this virus throughout more of North America [25,60]. The BTV-2 strain used in our study showed substantial genetic similarity to the 2010 California isolates of BTV-2 across all ten segments. BTV-2 is now considered enzootic in the U.S., but it has remained limited in its distribution. While underlying causes remain poorly understood, various factors including reduced vector competence of *C. sonorensis*—the predominant vector species of BTV in the U.S.—for BTV-2 likely contribute to this phenomenon [24,25,61]. Importantly, our finding of reassortment between BTV-2 and BTV-10 indicates that this virus has the ability to reassort with other enzootic strains of BTV, potentially allowing for expansion of a reassortant BTV-2 to areas where this virus has previously failed to circulate. Vector competence studies focusing on reassortant BTV-2 viruses are necessary to better estimate the risk that this poses.

Our findings are not only relevant to BTV ecosystems in North America, but also in other parts of the world where reassortment between established and newly introduced BTV strains continues to drive viral diversification. The relatively recent incursion of BTV-2 in Australia offers a contrasting example to the North American distribution of BTV-2: there, a BTV-2 strain that was first detected in 2008 subsequently reassorted extensively with enzootic strains [62]. These contrasting examples of BTV spread and reassortment highlight the complexities of global BTV ecosystems and underscore the importance of further studies.

Finally, metagenomic sequencing coupled with amplicon-based genotyping of plaques is a highly useful approach for investigating reassortment in a rapid, cost-efficient manner. Our findings highlight the utility of WGS in reassortment studies, especially when considering viruses with a relatively large number of segments, such as those in the family *Reoviridae*. BTV, with ten genome segments, can generate up to 1024 possible reassortants in the case of two co-infecting viruses. Robust analysis of population make-up during BTV coinfection using standard plaque assay-based methods thus requires thousands of individual plaques to be genotyped, rapidly becoming both labor- and cost-prohibitive in most cases. Even then, this premise is based on the assumption that all viable viruses generate plaques on the selected cell type, which is not necessarily valid. As such, WGS becomes a useful tool for detecting population-wide shifts in segment frequencies secondary to coinfection and provides a lens into potential reassortment events that arise, broadening our understanding of the global segment trends that accompany this important feature of segmented viruses.

## Figures and Tables

**Table 1:** Pairwise Identity of BTV-2 and BTV-10 across all ten segments (s1–s10) at the nucleotide and amino acid levels.

	S1	S2	S3	S4	S5	S6	S7	S8	S9	S10
% Nucleotide Identity	89.2%	52.4%	93.2%	89.7%	91.6%	69.4%	93.7%	89.3%	88.1%	81.9%
% Amino acid Identity	97.8%	40.9%	99.6%	96.4%	97.3%	77.9%	100%	90.4%	86.9%	93.9%

**Table 2:** Coding Sequence Coverage, All Samples. CDS = coding sequence.

		% CDS Coverage, BTV-2			% CDS Coverage, BTV-10			Total Reads, BTV-2			Total Reads, BTV-10			Mean CDS Depth, BTV-2			Mean CDS Depth, BTV-10		
Passage	Segment	2 + 10 A	2 + 10 B	2 + 10 C	2 + 10 A	2 + 10 B	2 + 10 C	2 + 10 A	2 + 10 B	2 + 10 C	2 + 10 A	2 + 10 B	2 + 10 C	2 + 10 A	2 + 10 B	2 + 10 C	2 + 10 A	2 + 10 B	2 + 10C
1	1	100%	100%	100%	100%	100%	100%	3655	2466	3461	1851	1240	1966	119	81	116	60	41	65
	2	100%	100%	100%	100%	100%	100%	2822	2023	4236	1509	1025	2190	124	90	192	66	47	99
	3	100%	100%	100%	100%	100%	100%	2677	1962	2661	1455	1104	1795	126	93	128	68	53	86
	4	99.9%	99.9%	99.5%	99.8%	99.7%	99.9%	1866	1392	1800	915	610	762	121	92	119	59	41	51
	5	100%	100%	100%	100%	100%	100%	3966	3046	4805	2129	1848	2512	299	259	368	161	141	193
	6	100%	100%	100%	100%	100%	100%	2697	2015	3793	1482	1153	1274	214	163	310	119	94	105
	7	100%	100%	100%	100%	100%	100%	2724	2371	2744	930	865	1191	323	282	329	110	105	144
	8	100%	100%	100%	100%	100%	100%	4028	3481	4660	1772	1414	1841	466	408	555	207	171	223
	9	100%	100%	100%	100%	100%	100%	4492	3474	4862	2324	1667	2256	550	430	621	282	208	286
	10	100%	100%	100%	100%	100%	100%	1577	1243	2036	1014	836	1156	274	221	371	188	158	217
3	1	100%	99.9%	100%	99.8%	97.8%	99.9%	1640	1453	1253	345	325	262	53	47	41	11	10	9
	2	100%	100%	100%	99.97%	99.9%	100%	1083	1024	921	355	309	246	48	45	41	16	14	11
	3	99.8%	100%	100%	99.90%	100%	100%	1093	1000	835	266	247	165	51	46	39	12	11	8
	4	99.8%	99.7%	99.0%	99.4%	95.8%	99.5%	903	952	795	138	146	129	59	62	52	9	10	9
	5	100%	100%	100%	99.4%	99.7%	100%	1718	1818	1589	914	1040	779	130	136	120	67	76	59
	6	100%	100%	100%	100%	100%	97.7%	1349	1575	1377	285	250	215	106	124	110	23	20	18
	7	100%	100%	100%	97%	100%	79.6%	1433	1824	1352	32	30	25	168	217	159	4	4	3
	8	100%	100%	100%	83.1%	71.5%	93.2%	2600	3081	2517	32	23	32	303	355	298	4	3	4
	9	100%	100%	100%	100%	100%	100%	2610	3123	2372	655	934	634	318	380	297	78	113	78
	10	100%	100%	100%	100%	100%	100%	596	725	593	331	435	327	102	127	106	62	82	59
7	1	100%	100%	98.6%	98.9%	99.9%	84%	664	1389	307	153	298	69	22	45	10	5	10	2
	2	100%	100%	100%	98%	97.8%	79.4%	509	961	216	112	223	65	22	42	10	5	10	3
	3	100%	100%	100%	80.9%	95.6%	57.9%	590	1099	231	61	108	37	28	51	11	3	5	2
	4	100%	100%	99.7%	91.3%	91.6%	48.7%	450	886	181	50	65	17	30	58	11	3	4	1
	5	99.6%	100%	100%	100%	100%	100%	357	645	157	455	706	184	26	48	11	34	53	14
	6	100%	100%	100%	100%	100%	100%	300	565	121	153	426	107	24	44	9	13	34	9
	7	100%	100%	100%	ND	21.7%	ND	538	852	261	ND	2	ND	59	97	28	ND	0	ND
	8	100%	100%	100%	ND	ND	ND	1018	1792	448	ND	ND	ND	118	210	50	ND	ND	ND
	9	99.8%	100%	100%	99.8%	100%	99.8%	224	537	105	207	479	111	27	65	13	25	59	13
	10	100%	100%	100%	100%	100%	99.4%	278	532	153	42	94	35	49	90	24	8	17	7

ND = not detected.

## Data Availability

The data presented in this study are available on request from the corresponding author.

## Supplementary Materials

The following are available online at https://www.mdpi.com/2076-2607/9/2/405/s1, Table S1: Amplicon Assay Primers, Table S2: Bowtie2 parameters used for amplicon analysis, Figure S1: Titers and Ct Values, Passages 1–7, Figure S2: Detection of each BTV segment via amplicon assay.
