# Supplementary material for: In Vitro Reassortment between Endemic Bluetongue Viruses Features Global Shifts in Segment Frequencies and Preferred Segment Combinations
Source: Microorganisms. 2021 Feb 16;9(2):405. doi: 10.3390/microorganisms9020405 (PMC7920030; doi:10.3390/microorganisms9020405)
Supplement: Supplementary file 1 [file microorganisms-09-00405-s001.pdf]

SUPPLEMENTAL MATERIALS

Supplemental Table S1 – Amplicon Assay Primers. Round 1 amplicon assay, BTV-specific primer sequences and concentrations. Final primer cocktail concentration (in µM) is shown for each primer.

	Sequence	µM
**S1_560F**	TCC AGG GGA ATA GAG ATT TAT C	0.12
**S1_1033R**	TCG TGC GAG CCY AAW TTT TG	0.12
**S2_1210F**	TGG CGA TGT KTA CTT YAC MTT GCG	0.11
**S2_1601R**	GCA TCY TTY TCG AAA TCG ATT GTA AG	0.11
**S3_2282F**	TMC AGT TYC GAG CGG CTT TAA G	0.10
**S3_2684R**	GAG CGA TTG GGT GAT GTC CA	0.10
**S4_1484F**	TCG TGG GCG ATG AAT TTT GCT	0.08
**S4_1961R**	TCA CCT AGC AGT CAC GCA TTA TAA G	0.08
**S5_177F**	TCG ATG ATY GCA GCA ACT GAT G	0.08
**S5_587R**	TGT GCT GTC CAC GAA TGC CAA	0.08
**S6_715F**	TAG GCG GCR TCW GAA GAA GTG	0.10
**S6_1099R**	YGG GAT CTT AAA YYT CAT CAT YAC	0.10
**S7_246F**	TTT TGG ACC GAT ATC GCC AGA	0.05
**S7_701R**	TGT CCA TCC CAC GCT ATA ATG C	0.05
**S8_594F**	TTG GAT GAW GAG GCC AAA GAG AT	0.09
**S8_1048R**	CTT AGA GAC AAA AGC AAC ACG CT	0.09
**S9_455F**	TAC GGT ACG AAG ATT GAT GTT TAC AG	0.09
**S9_902R**	TTC CAA TGC GGA TCT CCA GTT G	0.09
**S10_184F**	TAA ATY CTG GAC AAA GCG ATG TC	0.07
**S10_549R**	ACT YTT TGC GCA AAC CAT CAT CA	0.07

TruSeq adapter tags sit upstream of forward and reverse primers:

Forward: CTA CAC GAC GCT CTT CCG ATC [BTV-specific primer sequence, from table]
Reverse: CAG ACG TGT GCT CTT CCG ATC [BTV-specific primer sequence, from table]
Second round dual-index PCR primers.

i7 primer: CAA GCA GAA GAC GGC ATA CGA GAT [8-mer barcode] C AGA CGT GTG CTC TTC CGA TC
i5 primer: AAT GAT ACG GCG ACC ACC GAG ATC TAC ACG TTC TCT TAC A [8-mer barcode] CTA CAC GAC GCT CTT CCG ATC T

Supplemental Table S2. Bowtie2 parameters used for amplicon analysis.

```
cmd="bowtie2
 -x $bt_index
 -q
 -1 $f1
 -2 $f2
 -D 120
 -R 60
 -X 600
 --rg-id 1
 --rg SM:1
 --local
 --qc-filter
 --score-min C,160,1
 --no-unal
 --no-mixed
 --maxins 700
 --time
 --al-conc ${output_prefix}.conc_hits.fastq
 --threads 24
```

1. Langmead B, Salzberg SL. Fast gapped-read alignment with Bowtie 2. *Nat Methods*. 2012;9(4):357-359. doi:10.1038/nmeth.1923.

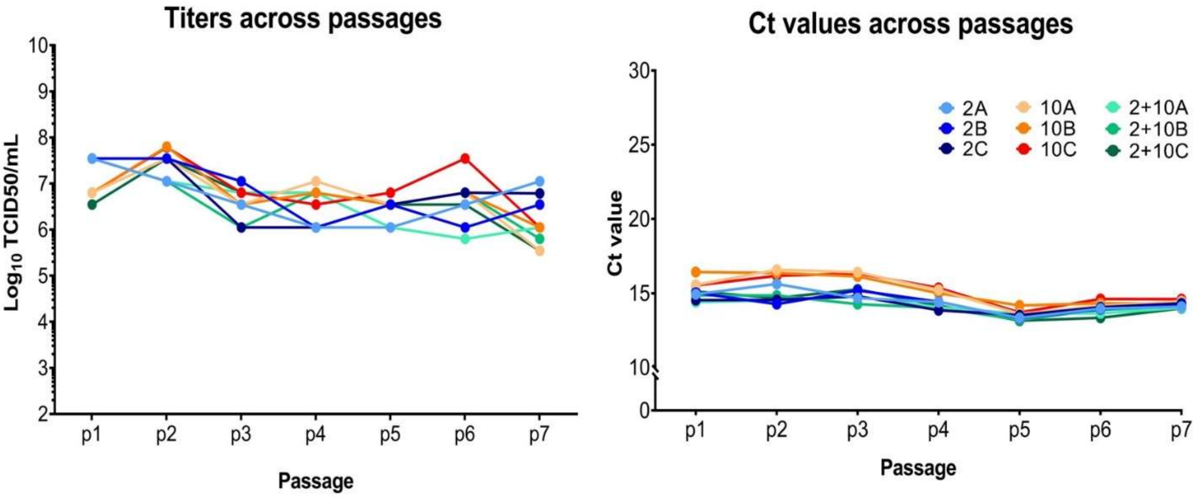

Supplemental Figure S1 – Titers and C_t Values, Passages 1-7. 50% tissue culture infectious dose (TCID50) across passages 1-7 and replicates is shown on the left hand panel, and BTV segment 10 C_t values across passages and replicates is shown on the right. Blue points and connecting lines indicate TCID50 and C_t value of BTV-2 replicates (2A, 2B, 2C). Orange/red points and connecting lines indicate TCID50 and C_t value of BTV-10 replicates (10A, 10B, 10C). Green points and connecting lines indicate TCID50 and C_t value of BTV-2+10 (coinfection) replicates (2+10A, 2+10B, 2+10C).

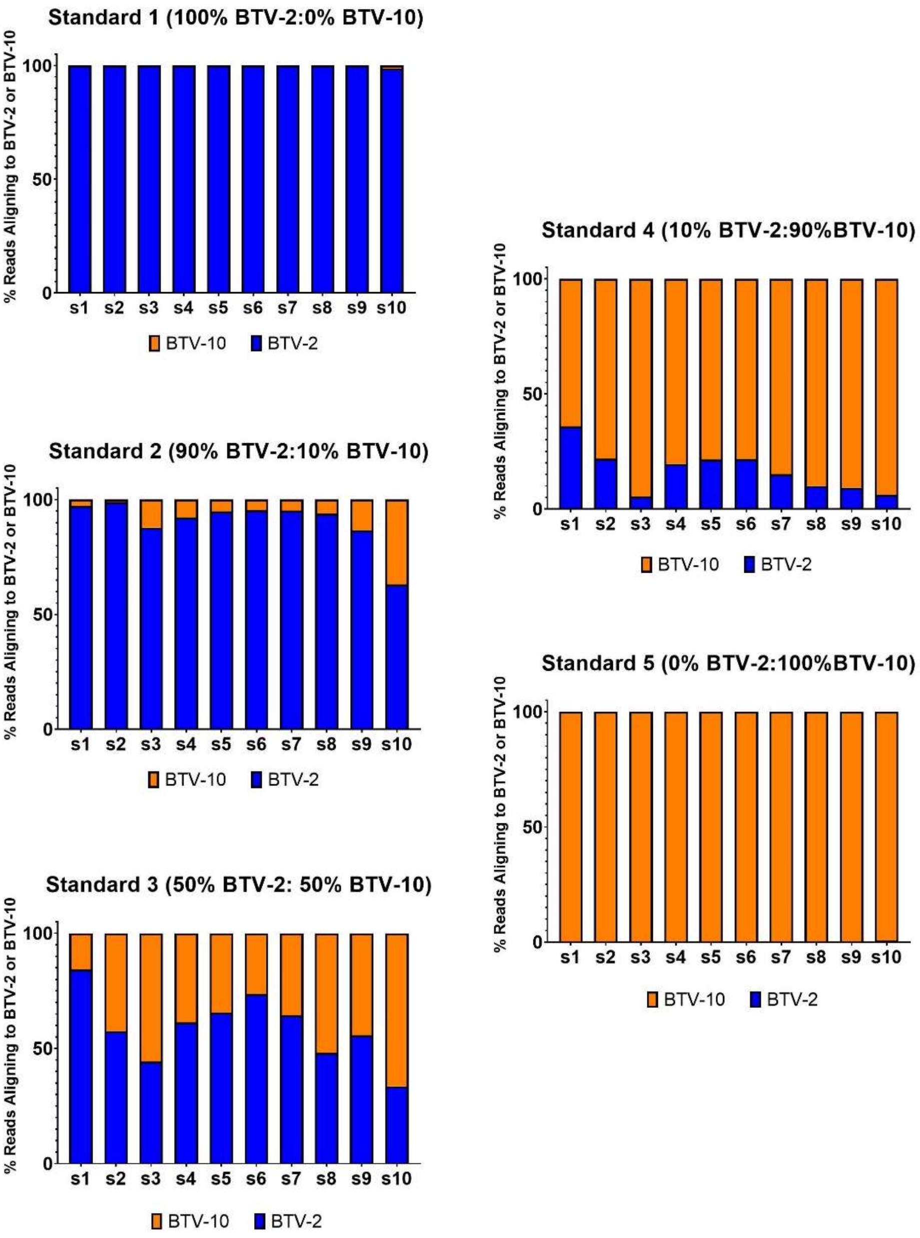

Supplemental Figure S2 – Detection of each BTV segment via amplicon assay. To establish the efficacy of the assay, 5 RNA standards were developed to demonstrate that the amplicon assay could distinguish between each segment for BTV-2 and BTV-10. Standards were prepared from BTV-2 and BTV-10 RNA stocks with equal Ct values (based on RT-PCR) as follows: standard 1, 100% BTV-2; standard 2, 90% BTV-2:10% BTV-10; standard 3, 50% BTV-2:50% BTV-10; standard 4, 10% BTV-2:90% BTV-10; standard 5, 100% BTV-10. Standards 1-5 were run alongside each amplicon assay. The percentage of amplicon reads aligning to each BTV-2 or BTV-10 segment (shown in blue and orange, respectively) for each standard are shown. Percentage data represent the mean from standards run across multiple amplicon assays (n = 9 or 10 replicates per each standard).
